# Genotyping single point mutations in rd1 and rd8 mice using melting curve analysis of qPCR fragments

**DOI:** 10.1038/s41598-024-70949-8

**Published:** 2024-08-28

**Authors:** Melanie E. Schwämmle, Felicitas Bucher, Günther Schlunck, Gottfried Martin

**Affiliations:** 1 Eye Center, Medical Center, Faculty of Medicine, University of Freiburg, Killianstr. 5, 79106 Freiburg, Germany; 2 Faculty of Biology, University of Freiburg, Freiburg, Germany

**Keywords:** ARMS PCR, Genotyping, Single nucleotide variant, Biological techniques, Genetics, Molecular biology, Biomarkers, Neurology

## Abstract

PCR is tolerant to single nucleotide mismatches. Therefore, genotyping of point mutations by PCR requires special conditions for the amplification of allele-specific PCR fragments. MS-PCR (mutagenically separated PCR) is an improved version of ARMS (amplification refractory mutation system) in which additional nucleotide mismatches near the mutation site are used to separate the wt fragments from the mutant fragments in a single-tube PCR. In the originally described procedure, the resulting fragments are resolved on agarose gels according to differences in size introduced by different lengths of the allele-specific primers. In order to evaluate the PCR fragments by melting curve analysis, we enlarged the difference in the melting temperatures of the fragments of the two alleles by increasing the GC content of the longer allele-specific primer resulting in a higher melting temperature of the corresponding fragment. Using the murine retinal degeneration mutations rd1 and rd8 as an example, we show that such primers result in an easy to handle genotyping procedure: qPCR followed by melting curve analysis. In summary, MS-PCR is a simple and easy-to-use method for detecting single nucleotide variants.

## Introduction

Many diseases such as haemophilia, Duchenne muscular dystrophy, or retinal degeneration are caused by mutations of single nucleotides. Such diseases are rare but the effects are often very destructive to the patients. They are investigated in animal models that have the same point mutation in the corresponding gene, and a prerequisite is that the animals have to be genotyped correctly before developing a phenotype. Therefore, simple and reliable detection methods are required.

**Fig. 1:**
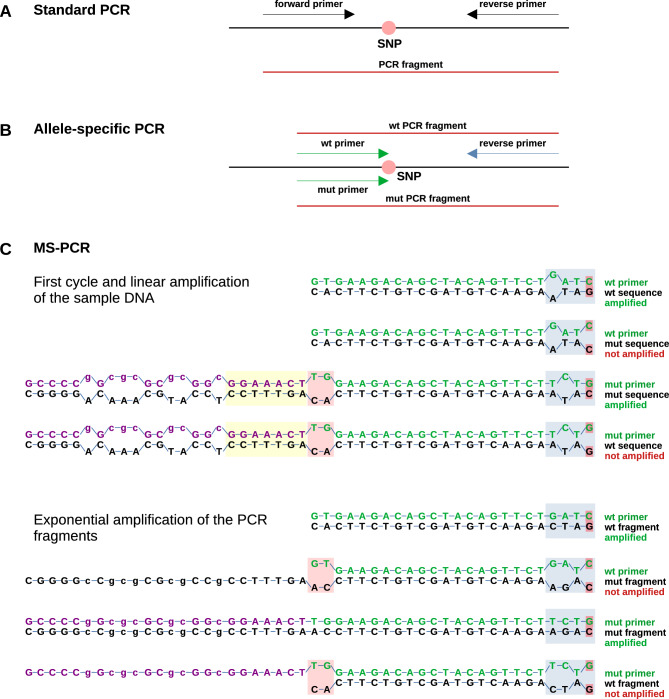
Principle of MS-PCR. (**A**) Standard PCR for evaluation via RFLP, high resolution melting curve analysis, or sequencing showing primers (arrows) and mutation site (SNP). (**B**) Allele-specific PCR for evaluation via agarose gel electrophoresis, melting curve analysis, or fluorescent labels showing primers (arrows) and mutation site (SNP). (**C**) MS-PCR is explained for a het rd8 mouse with the primers Crb1-mF1 (wt) and Crb1-mF2 (mut). The mutated base is indicated by a small red box. In the first PCR cycle and in the linear amplification with the original DNA fragments, the wt primer binds to the wt sequence of the original DNA fragment with one mismatch and to the mut sequence with two mismatches at the 3ʹ end (blue box), while the mut primer binds to the mut sequence with one mismatch and to the wt sequence with two mismatches at the 3ʹ end. In the following cycles of exponential amplification, the wt primer will match exactly to the wt fragment and the mut primer will match exactly to the mut fragment while the wt primer will have three mismatches with the mut fragment and the mut primer will have three mismatches with the wt fragment at the 3ʹ end (blue box), preventing amplification under appropriate conditions. The two mismatches of the mutant primer with the original DNA sequence at the 5ʹ end (red box) reduce the stability of the unwanted combinations. The distance between the blue and red boxes affects the stability of the primer annealing and should be about 18 bp. In addition, the 6–7 bp of the mut primer left to the red box (yellow box) should match the wt sequence exactly in order to stabilize the annealing of the primer during the first cycle and linear amplification.

Genotyping of single nucleotide substitutions (SNPs, single nucleotide polymorphisms) can be achieved by different methods^1^. Most of them are based on polymerase chain reactions (PCR). In standard PCR procedures using one forward and reverse primer pair, the amplified fragment is evaluated by various methods such as digestion with a restriction enzyme that is sensitive to the mutated site (RFLP, restriction fragment length polymorphism), sequencing the PCR fragment, or high resolution melting analysis^2,3^ (Fig. 1A). In other PCR based methods, two allele-specific forward and one reverse primers are used to produce specific fragments for different alleles (allele-specific PCR (AS-PCR)) followed by the evaluation of the PCR products in different ways such as agarose gel electrophoresis or various kinds of fluorescent labels (Fig. 1B).

In contrast to AS-PCR targeting mutations caused by insertions or deletions of larger DNA fragments, detecting single point substitutions by AS-PCR faces two challenges: (1) PCR is tolerant to single point variations, and, thus, a single base mismatch does not necessarily prevent primer binding and elongation. (2) Both allele-specific primers must include the mutated site and, consequently, have to bind at the same site (Fig. 1B). Therefore, a specialized variant of allele-specific amplification must be used that increases the difference caused by the mutation to yield two different fragments from the mutated (mut) and wild-type (wt) DNA strands. Several methods of this AS-PCR type were developed such as ARMS (amplification refractory mutation system)^4,5^ or Melt-MAMA (melt analysis of mismatch amplification mutation assays)^6^, that rely on an additional mismatch base introduced at the penultimate or antepenultimate position at the 3ʹ end of the primer. PCR conditions must be carefully controlled to get reliable results.

**Fig. 2:**
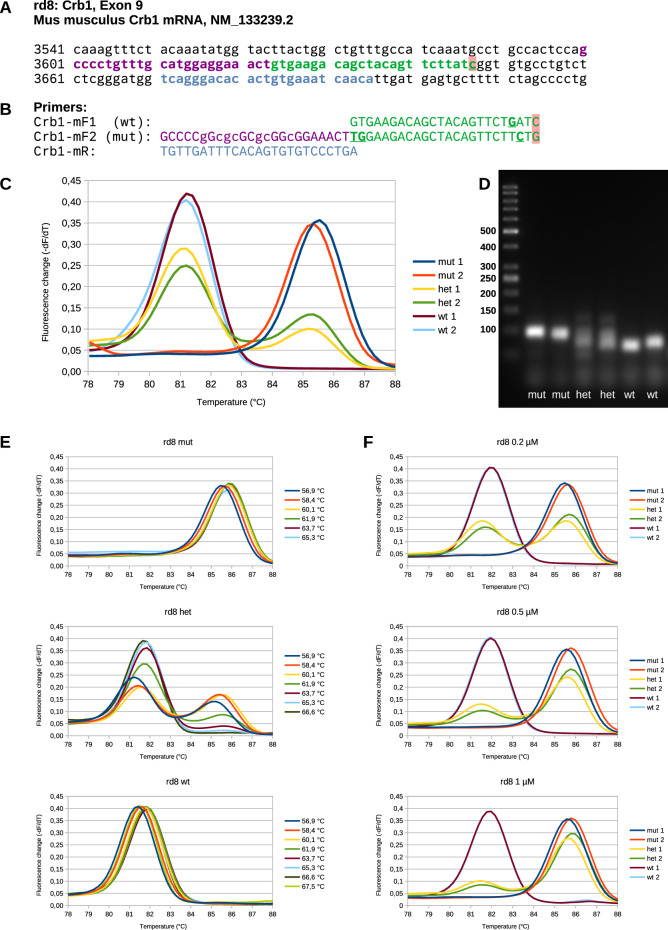
Genotyping rd8 in exon 9 of Crb1. (**A**) Part of Crb1 sequence including the mutated site at C3647G (red). Primer positions are indicated in the same color as in (**B**). (**B**) Primer mF1 amplifies the wt fragment while mF2 amplifies the mutated fragment. Both contain additional nucleotide changes (bold and underlined) to enhance strand specificity. The violet part was added to get a size difference in the PCR product for analysis by agarose gel electrophoresis, and its sequence was changed to enhance GC content and melting temperature. (**C**) Melting curve analysis showing two different peaks for wt and mut, resp., while het shows both peaks. Two samples of each genotype are shown. (**D**) Agarose gel showing the same PCR fragments as in C. Fragment sizes: 72 bp (wt) and 96 bp (mut). (**E**) Melting curves of one sample of each genotype at different annealing temperatures. While the curves of mut and wt samples are not sensitive to temperature, those of het samples show that the mut peak disappears at high temperatures underlining the importance of using a temperature of 60 °C. (**F**) Melting curve analysis of samples run with different concentrations of primer Crb1-mF2. A concentration of 0.2 µM resulted in equal height of mut and wt peaks in samples of het mice.

**Fig. 3:**
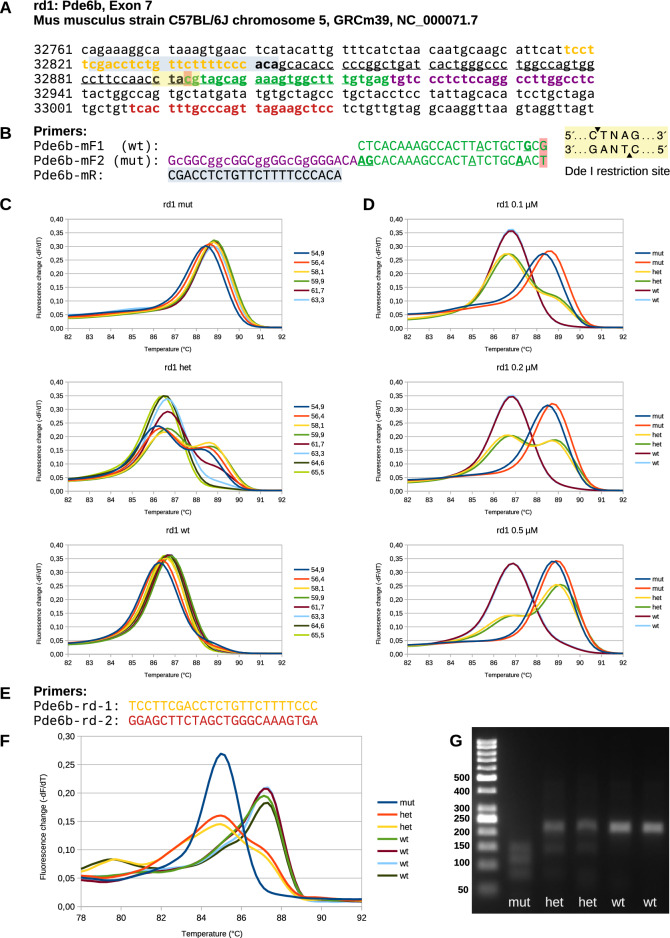
Genotyping rd1 in exon 7 of Pde6b. (**A**) Genomic Pde6b sequence including the mutated site at C32893A (Y347stop, red). Exon 7 is underlined. Primers are indicated in the same color as in (**B**) and (**E**). The yellow box indicates the Dde I restriction site. (**B**) Primer mF1 amplifies the wt fragment while mF2 amplifies the mutated fragment. Both contain additional nucleotide changes (bold and underlined) to enhance strand specificity. Two additional changes (underlined but not in bold) were introduced to reduce the formation of primer dimers. The violet part was added to get a size difference, and its sequence was changed to enhance GC content and melting temperature. Fragment sizes: 95 bp (wt) and 119 bp (mut). Note that the primers are indicated for the complementary strand. (**C**) Melting curves at different annealing temperatures. While the curves of mut and wt samples are not sensitive to temperature, those of het samples are highly sensitive, highlighting the importance of using a temperature of 60 °C. (**D**) Melting curve analysis of samples run with different concentrations of primer Pde6b-mF2. A concentration of 0.2 µM resulted in equal height of mut and wt peaks in samples of het mice. (**E**) Analysis of the rd1 mutation using a single PCR fragment followed by Dde I digestion as described in the original publication^10^. The primers span the mutation site as shown in (**A**). (**F**) Melting curve analysis of a fragment amplified with the primers shown in (**E**) revealing different shapes for the wt, het, and mut fragments after digestion with Dde I. (**G**) Agarose gel showing the same PCR fragments as in F that were cut with Dde I. Fragment sizes: 214 bp (wt), 140 bp (mut), 77 bp (mut).

MS-PCR (mutagenically separated PCR) is an improved variant of AS-PCR for SNPs^7^. Here, the enhanced difference between the different alleles is obtained by introducing more than one additional base mismatch in both the wt and mut primer sequences that suppress the amplification of the wt allele with the mut primer and the mut allele with the wt primer (Fig. 1C). MS-PCR applies two allelic primers that differ at three positions within the four nucleotides at the 3ʹ end (including the single point mutation) and additionally at two nucleotides of the longer primer corresponding to the 5ʹ end of the shorter primer (Fig. 1, Fig. 2A and B, Fig. 3A and B)^7^. Originally, one of the two allele-specific primers of MS-PCR has an extension that is used to introduce a size difference for evaluation on agarose gels. However, this extension can also be modified to introduce a shift in the GC content for melting curve analysis^8,9^. A replacement of A and T with C and G or an artificial GC clamp results in a higher melting temperature. This simple principle was applied in the present study. As an example, the point mutations rd1 and rd8 that result in retinal degeneration in mice were investigated. We found that MS-PCR for rd1 and rd8 is a simple, robust, and inexpensive method for genotyping.

## Materials and methods

### Ethical statement

No single mouse was killed for this study. All mice were used in other projects that needed genotyping and that adhered to the animal care guidelines of the Institute for Laboratory Animal Research (Guide for the Care and Use of Laboratory Animals) in accordance with both the ARRIVE guidelines and the ARVO Statement for the Use of Animals in Ophthalmic and Vision Research and that were approved by the local animal welfare committee (Tierschutzkommission).

### DNA extraction

DNA was extracted by a standard protocol from Jackson (Jackson Laboratory, Bar Harbor, Maine, USA). One ear punch (around 1 mm^2^) of a mouse ear was placed in a 0.5 ml reaction tube containing 75 µl of 25 mM NaOH/0.2 mM EDTA. After heating at 98 °C for 30 min, the solution was neutralized by adding 75 µl of 40 mM Tris HCl (pH 5.5) and centrifuged at 13,000 rpm (13,600×*g*) for 3 min. The supernatant was used for PCR.

### PCR

1 µl of the extracted DNA solution was used in a 20 µl PCR reaction mix containing 10 µl of a 2× SYBR Green PCR buffer (TB Green^®^ Premix Ex Taq^™^ Tli RNaseH Plus, RR420L, Takara Bio Europe, Saint-Germain-en-Laye, France) and 2 µl of a primer mix (rd1: 0.5 µM Pde6b-mF1 (CTCACAAAGCCACTTACTGCTGCG), 0.2 µM Pde6b-mF2 (GcGGCggcGGCggGGcGgGGGACAAGCACAAAGCCACTATCTGCAACT), 0.5 µM Pde6b-mR (CGACCTCTGTTCTTTTCCCACA); rd8: 0.5 µM Crb1-mF1 (GTGAAGACAGCTACAGTTCTGATC), 0.2 µM Crb1-mF2 (GCCCCgGcgcGCgcGGcGGAAACTTGGAAGACAGCTACAGTTCTTCTG), 0.5 µM Crb1-mR (TGTTGATTTCACAGTGTGTCCCTGA)). After pre-heating in a LightCycler 96 (Roche Diagnostics, Mannheim, Germany) at 95 °C for 30 s, a two-step amplification with 35 cycles of melting at 95 °C for 10 s, annealing and elongation at 60 °C for 60 s was followed by recording of a melting curve from 65 to 97 °C. Every test included known mut, heterozygous (het), and wt samples as a standard.

In addition, the genotyping results for rd1 were checked with the RFLP assay described in the first publication^10,11^. A PCR fragment including exon 7 of the Pde6b gene (rd1) was amplified using the primers Pde6b-rd-1 (TCCTTCGACCTCTGTTCTTTTCCC) and Pde6b-rd-2 (GGAGCTTCTAGCTGGGCAAAGTGA) under the same conditions as described above. After PCR, 1 µl of RE buffer and 1 µl (10 units) of Dde I (R0175S, New England Biolabs, Frankfurt am Main, Germany) were added and the mixture was incubated at 37 °C for 1 h. The resulting fragments were investigated by melting curve analysis.

## Results

MS-PCR for the rd8 mutation in the murine Crb1 gene was described with evaluation on agarose gels^12,13^. This method was employed to determine the rd8 genotype of mice used in the subsequent experiments. Unfortunately, the fragments for mut and wt had the same melting temperature in the SYBR green qPCR. Therefore, we modified the end of the longer primer by replacing most of the A and T by C and G resulting in a shift in the melting temperature to be detectable in the melting curve of the qPCR fragments (Fig. 2A and B, mut, small letters). The difference of the melting points between the mut and wt fragments depends on the length (they should be short) and the GC content of the amplified fragments. For good separation, the length of the GC clamp must be adjusted.

Melting curve analysis of MS-PCR for rd8 showed two distinct peaks at 81.5 °C (wt) and 85.7 °C (mut, Fig. 2C) that clearly indicated the genotype of the sample. Some samples were checked by agarose gel electrophoresis after qPCR (Fig. 2D) confirming the genotyping results obtained by melting curve analysis. The melting curves of het mice were sensitive to the annealing and amplification temperature (Fig. 2E), which must be carefully controlled. A temperature of 60 °C worked well. The relative height of the two peaks in heterozygotes was adjusted by changing the concentration of the primer Crb1-mF2 (Fig. 2F). A dilution range from 60 to 0.5 µg of template DNA was suitable for PCR (Supplementary Fig. 1).

In addition, we designed a respective primer system for the murine rd1 mutation in the Pde6b gene (Fig. 3A, B). Already small changes in the sequence of the mutant primer lead to large differences in the results (Supplementary Fig. 2). However, after adjusting the primer sequence, equivalent results were obtained for the rd1 mutation (Fig. 3). The melting curves showed peaks at 86.8 °C for wt and at 88.8 °C for the mut fragment (Fig. 3C, D) which corresponded to the respective fragments detected by agarose gel electrophoresis (data not shown). Due to the higher melting temperature of the wt fragment compared to the rd8 wt fragment the difference between the wt and the mut fragments was smaller. The optimum for the annealing and amplification temperature as well as for the concentration of primer Pde6b-mF2 were equal to those found for rd8.

Finally, rd1 genotyping was verified by the original method for genotyping the rd1 mutation^10,11^ involving restriction enzyme digestion with Dde I of a single PCR product (RFLP, Fig. 3E–G). Melting curve analysis revealed different peaks for wt and mut fragments (Fig. 3F). Agarose gel electrophoresis confirmed the result. The fragment of 214 bp (base pairs) was wt while the fragments of 140 bp and 77 bp indicated the cleaved mut fragments (Fig. 3G). Dde I digestion was also possible with the MS-PCR fragments as described above but the peaks in the melting curves were not well separated, thus providing no additional information (Supplementary Fig. 3). Finally, the results were verified by sequencing the amplified single rd1 fragment (without Dde I digestion, data not shown). The results corresponded well with those of the MS-PCR confirming that the latter is a valid tool for rd1 genotyping.

## Discussion

Immediately after the invention of PCR, researchers sought for methods to utilize it for detecting single nucleotide mutations. One possibility is simply to sequence the amplified DNA fragment. Another possibility is to cut the amplified DNA fragment with a specific restriction enzyme whose recognition site is located at the SNP (RFLP). Other methods integrated the SNP into the primer sequence to create allele-specific primers (AS-PCR). ARMS^4,5^, a wide-spread version of this technique, uses an additional mutation at the penultimate base at the 3ʹ end of the allele-specific primers. Unfortunately, the mut primer may bind to the wt fragment and the wt primer to the mut fragment. One way to resolve this problem is to amplify the wt fragment and the mut fragment in separate tubes. However, optimized PCR conditions, including improved primer design, allow this type of AS-PCR to be performed in a single tube.

Another solution to suppress the amplification of the wt fragment with the mut primer or the mut fragment with the wt primer is MS-PCR that uses additional mutations near the 3ʹ and 5ʹ ends of the primers. This strategy has the potential to suppress the annealing of the mut primers to the wt fragments and of the wt primers to the mut fragments, resulting in a stable separation of mut and wt fragments. These mismatches require a specific annealing temperature in the first cycles to prevent unspecific binding, and this temperature has to be carefully controlled.

Several studies have reported the use of MS-PCR for selected SNPs, and some have combined it with melting curve analysis as in our study^8,9^. This results in an easy-to-use method that can be used to optimize MS-PCR by defining the optimal primer concentrations or annealing temperatures as well as by searching for the best primer parameters (length and additional mutations). MS-PCR would greatly benefit from a systematic investigation of effective base substitutions, similar to the results reported for ARMS^5^. In addition, a software tool for MS-PCR primer design similar to WASP, that allows the design ofARMS primers, would facilitate the workflow^14^.

Fragments amplified in AS-PCR can be analyzed in different ways. In the past, they were analyzed by agarose gel electrophoresis. One of the primers contains an extension of 20–30 base pairs resulting in a detectable size difference. In fact, such an extension can also be used for other detection methods. Melt-MAMA^6^ is a SNP detection method using allele-specific primers labeled with a GC clamp for analysis of the PCR products by melting curves^15^. However, allele-specific primers labeled with FRET probes are more common. Large commercial platforms using this technique are KASP^16^ and rhAmp^17^.

In general, MS-PCR combined with melting curve analysis is a simple, reliable, and affordable method for genotyping single nucleotide substitutions from any source, with the potential to be scaled up for high-throughput studies.

### Supplementary Information

Supplementary Figures.

## Data Availability

Data is provided within the manuscript or supplementary information files. Data used and analysed during the current study to optimize the procedures are available from the corresponding author on reasonable request. The authors declare no competing interests.
