# Supplementary material for: Genotyping single point mutations in rd1 and rd8 mice using melting curve analysis of qPCR fragments
Source: Sci Rep. 2024 Aug 28;14:19909. doi: 10.1038/s41598-024-70949-8 (PMC11358275; doi:10.1038/s41598-024-70949-8)
Supplement: Supplementary file 1 — Supplementary Figures. [file 41598_2024_70949_MOESM1_ESM.pdf]

Suppl. Fig. 1. Effect of DNA dilution.

DNA concentrations in a 100-fold dilution range are suitable for rd8 detection by MS-PCR.

Suppl. Fig. 2. Influence of different primer sequences on the performance of MS-PCR for rd1.

Gradient PCR followed by melting curve analysis was used to investigate the performance of different mut primers. As a result, the distance between the mismatches at the 5' and the 3' ends of the mut primers should be around 18 bp. The 6 - 7 bp upstream of the 5' end mismatches should be exact matches to stabilize the primer annealing. The wt primer was CTCACAAAGCCACTTACTGCTGCG for Pde6b-mF2 and CAAAGCCACTTACTGCTGCG for the others. CGACCTCTGTTCTTTTCCCACA was used as the reverse primer.

Suppl. Fig. 3. Dde I digestion after MS-PCR for rd1.

The differences between the melting curves of the mut and wt fragments are small so that the het signal is hard to detect.

Suppl. Fig. 1

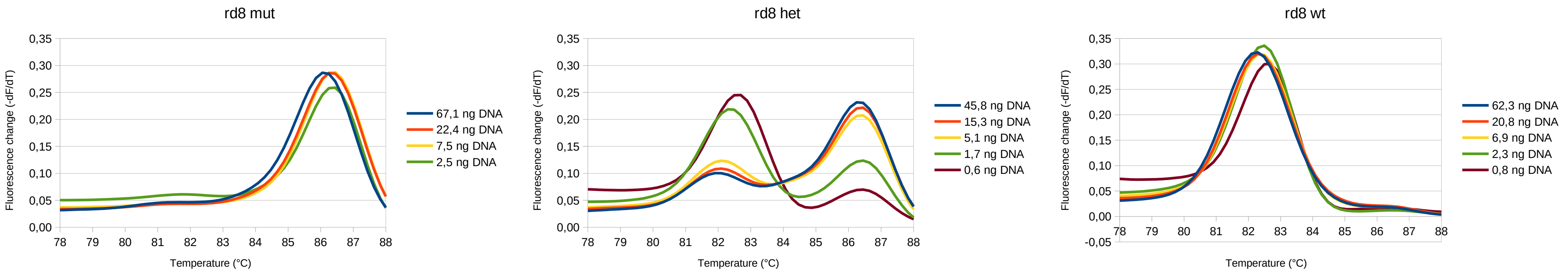
rd8 mut
Fluorescence change (-dF/dT)
0,35
0,30
0,25
0,20
0,15
0,10
0,05
0,00
78 79 80 81 82 83 84 85 86 87 88
Temperature (°C)
67,1 ng DNA
22,4 ng DNA
7,5 ng DNA
2,5 ng DNA
rd8 het
Fluorescence change (-dF/dT)
Temperature (°C)
45,8 ng DNA
15,3 ng DNA
5,1 ng DNA
1,7 ng DNA
0,6 ng DNA
rd8 wt
Fluorescence change (-dF/dT)
-0,05
Temperature (°C)
62,3 ng DNA
20,8 ng DNA
6,9 ng DNA
2,3 ng DNA
0,8 ng DNA

Suppl. Fig. 2

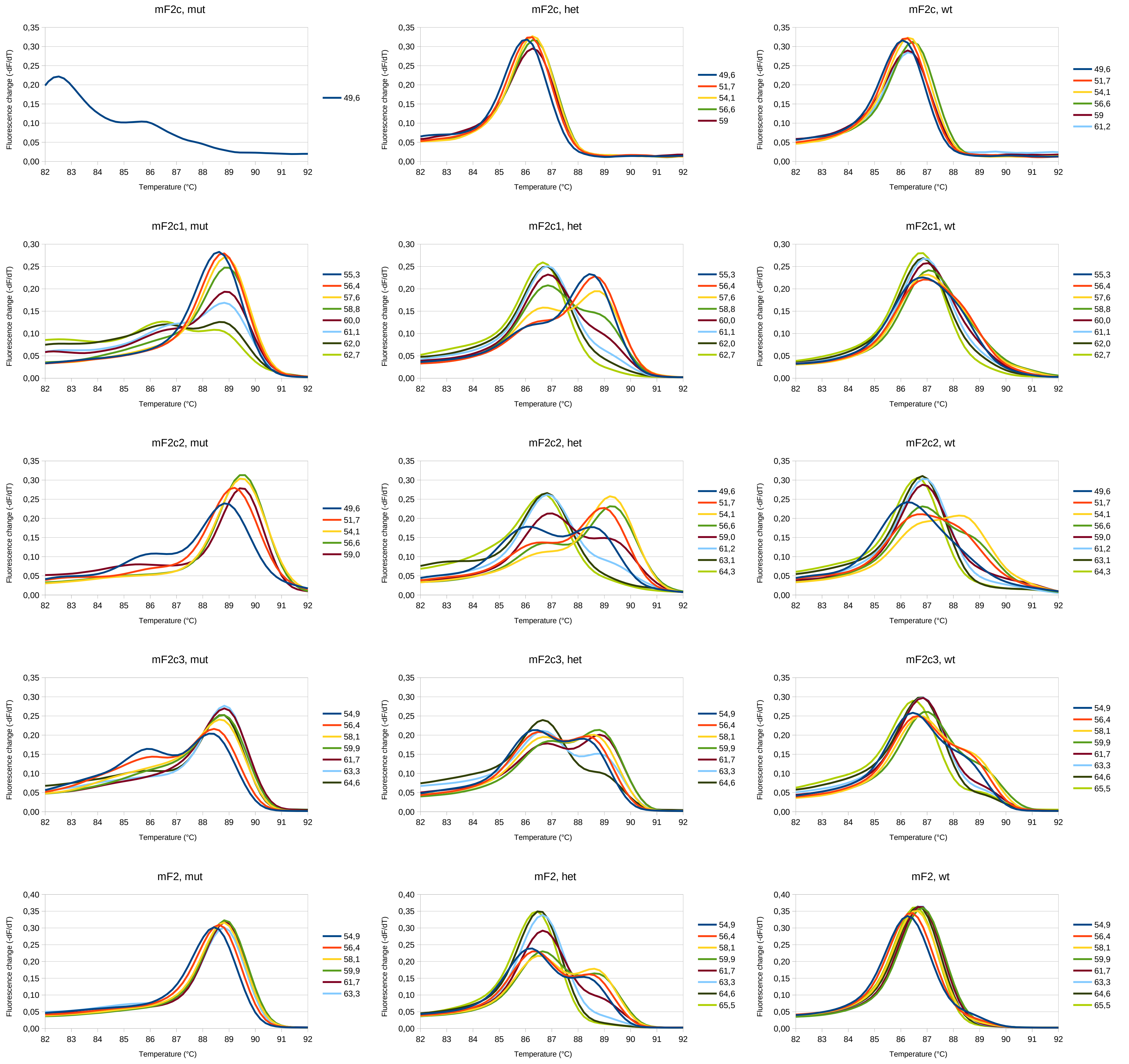


```
Pde6b-mF2c:                CggcGGCggGGcGgGGGcCgggCATCAAGCCACTATCTGCAACT
Pde6b-mF2c1:             GGCggcGGCggGGcGgGGGcCACTCATCAAGCCACTATCTGCAACT
Pde6b-mF2c2:           GcGGCggcGGCggGGcGgGGGcCACTCATCAAGCCACTATCTGCAACT
Pde6b-mF2c3:           GcGGCggcGGCggGGcGgGGGACACTCATCAAGCCACTATCTGCAACT
Pde6b-mF2:             GcGGCggcGGCggGGcGgGGGACAAGCACAAAGCCACTATCTGCAACT
DNA sequence: CTGGCCAGTAGAGGCCAAGGCCTGGAGAGGGACACTCACAAAGCCACTTTCTGCTACGTAGGTT
```

Suppl. Fig. 3

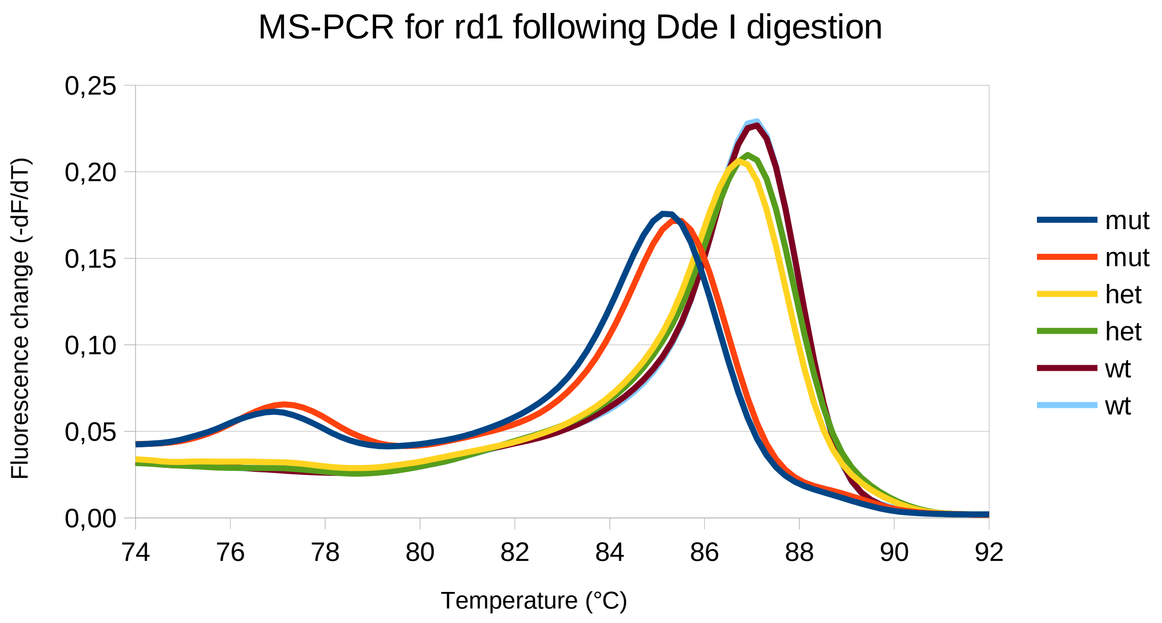
MS-PCR for rd1 following Dde I digestion
Fluorescence change (-dF/dT)
0,25
0,20
0,15
0,10
0,05
0,00
74
76
78
80
82
84
86
88
90
92
Temperature (°C)
mut
mut
het
het
wt
wt
